# Reward processing in children with affective dysregulation

**DOI:** 10.1002/jcv2.70102

**Published:** 2026-03-07

**Authors:** Pascal‐M. Aggensteiner, Francisca Giller, Nathalie Holz, Anna Kaiser, Christine Igel, Sarah Hohmann, Manfred Döpfner, Veit Roessner, Christian Beste, Tobias Banaschewski, Daniel Brandeis, the ADOPT Consortium

**Affiliations:** ^1^ Department of Child and Adolescent Psychiatry and Psychotherapy Central Institute of Mental Health Medical Faculty Mannheim/Heidelberg University Mannheim Germany; ^2^ German Center for Mental Health (DZPG) partner site Mannheim‐Heidelberg‐Ulm Germany; ^3^ Department of Cognitive Neurophysiology Department of Child and Adolescent Psychiatry Faculty of Medicine Technische Universität Dresden Dresden Germany; ^4^ Department of Child and Adolescent Psychiatry and Psychotherapy and Psychosomatics University Medical Center Hamburg‐Eppendorf Hamburg Germany; ^5^ Department of Child and Adolescent Psychiatry, Psychosomatics and Psychotherapy Medical Faculty University of Cologne Cologne Germany; ^6^ German Center for Child and Adolescent Health (DZKJ) partner site Leipzig‐Dresden Germany; ^7^ Department of Child and Adolescent Psychiatry and Psychotherapy University Hospital of Psychiatry Zurich University of Zurich Zurich Switzerland; ^8^ Neuroscience Center Zurich University and ETH Zurich Zurich Switzerland

**Keywords:** EEG, emotion dysregulation, irritability, reward anticipation

## Abstract

**Background:**

Affective dysregulation (AD) in children is characterized by irritability, anger, and frequent intense temper outbursts. Considerable evidence implies altered processing of frustration about missed rewards, but few studies investigated the preceding and thus potentially predictive reward anticipation and initial delivery processing in children with AD.

**Methods:**

A total of 103 children aged 8–12 years (50 with AD and 53 without AD) were examined during a monetary reward anticipation task with event‐related potential (ERP) components resolving reward anticipation (cue‐CNV [Contingent Negative Variation]) and reward delivery phases (Reward Positivity and Feedback‐Related Negativity). All components were analyzed by repeated measures analysis of variance. Regression analyses also evaluated the associations between those ERP components and dimensional AD symptoms.

**Results:**

Children with AD showed attenuated anticipatory reward processing compared to No‐ADs. The CNV at fronto‐central site (FCz) showed a significant group effect (No‐AD > AD, *p* = 0.017). Post‐hoc test showed that this group difference was stronger for the cue monetary condition (monetary cue: *p* = 0.007, *d* = 0.56, verbal cue: *p* = 0.901, *d* = 0.16), and that only the No‐AD group showed a significant difference between conditions (*p* < 0.001). No significant effects were obtained for the delivery phase. Regression analysis showed that a reduced anticipatory CNV at FCz significantly explained AD symptoms, and that anger/irritability and anxiety/depressive symptoms predicted a reduced anticipatory CNV at FCz.

**Conclusion:**

This neurophysiological characterization of reward anticipation and delivery in children with AD demonstrates altered neural activity in AD during anticipation of reward rather than following the delivery (or omission) of the reward itself. Our results highlight that altered reward anticipation in AD can occur outside frustration‐prone tasks or settings, and underline the important role of both anger/irritability and anxiety/depressive symptoms in the pathophysiology of AD for atypical reward anticipation.

## Key Points

**What's known?**

- Children with Affective dysregulation (AD) exhibited reduced anticipatory reward processing compared to those without AD.

**What's new?**

- No significant differences were observed during the reward‐delivery phase.
- Blunted reward anticipation was associated with symptoms of anger/irritability and anxiety/depression.

**What's relevant?**

- Identifying deficits in reward anticipation could help refine diagnostics and develop more targeted therapies.

## INTRODUCTION

Anger, aggression and irritability are key elements of affective dysregulation (AD) in children. AD is characterized by a general irritable mood plus age‐and‐situation‐inappropriate temperament outbursts with aggressive tendencies (Brotman et al., 2017). While the terms irritability and AD are frequently used interchangeably in both clinical and research settings (Döpfner et al., 2019; Waltereit et al., 2019), it is important to recognize that AD represents a wider construct. The present report from our large multicenter study (ADOPT: AD—Optimizing Prevention and Therapy) uses this broader conceptualization of AD. Specifically, while irritability—defined primarily as a heightened proneness to anger—is one component of AD (Stringaris & Taylor, 2015), AD also encompasses a wider range of dysregulated emotional responses, including anxiety, sadness, and even excessive positive affect such as exuberance (Treier et al., 2023). This broader definition aligns with growing recognition of AD as a transdiagnostic construct that spans multiple psychiatric disorders, including disruptive mood dysregulation disorder (DMDD), oppositional defiant disorder (ODD), conduct disorder (CD), and attention‐deficit/hyperactivity disorder (ADHD) (Ambrosini et al., 2013; Jensen et al., 2007; Rich et al., 2011; Shaw et al., 2014).

Altered reward processing has been proposed as a key mechanism of irritability (Brotman et al., 2017) or AD (Döpfner et al., 2019). Although changes in reward processing with AD have been described (Avenevoli et al., 2015; Kessel et al., 2016; Leibenluft & Stoddard, 2013), this research mainly focused on the delivery phase during frustrating situations where expected rewards are not received, aligning with the frustrative non‐reward, rather than the reward anticipation construct also listed in the Research Domain Criteria (RDoC) (Insel et al., 2010; Musser & Raiker, 2019). Within the RDoC framework, reward processing falls under the Positive Valence Systems domain, which includes anticipatory (reward anticipation) and early outcome‐related processes (such as reward responsiveness). Accordingly, the present study focuses on reward processing as a complementary construct in children with AD. Several EEG studies, although mainly in healthy controls or community samples, demonstrated significant electrophysiological alterations with AD‐related symptoms, such as irritability and emotion regulation difficulties, during the absence of an expected reward (frustrative non‐reward, i.e., Deveney et al., 2019; Waxmonsky et al., 2019) and the receipt of a rewarding stimulus (reward sensitivity, i.e., Kessel et al., 2016; Waxmonsky et al., 2022). However, the findings regarding the reward delivery phase remain inconsistent, indicating either hypo‐ or hyperactivity, which is likely partially attributed to the heterogeneous methodology (Hennefield et al., 2022) and definitions of AD‐symptoms across studies.

Surprisingly, no event‐related potential (ERP) studies have yet assessed whether AD in groups meeting stringent criteria is associated with reward anticipation preceding reward attainment or frustration, although reward anticipation is a core aspect of reward processing, an established RDoC construct, and known to partially predict reward processes in healthy controls (Zubovics et al., 2021). Our study therefore, specifically investigates anticipatory reward processes in well‐characterized young children with AD.

Reward anticipation is typically measured in reward processing tasks by the CNV, a slow cortical potential linked to anticipation, expectation and cognitive preparation in a wide range of tasks. This CNV builds up approximately one second after a reward cue until the reward target appears, precedes feedback, and is stronger (more negative) in response to the anticipation of more salient rewards (Boecker et al., 2014; Broyd et al., 2012). It is probably generated in the dorsal anterior cingulate, frontal cortex and midbrain dopaminergic nuclei (Albrecht et al., 2013; Plichta et al., 2013), susceptible to dopaminergic modulation (Linssen et al., 2011). Studies assessing reward anticipation reported that participants self‐reporting more difficulties in regulating emotions showed weaker anticipatory ERPs during a monetary incentive delay task (Zubovics et al., 2021) in high school students and in adults with depressive symptoms (Ait Oumeziane et al., 2019).

Regarding the components related to reward delivery, the Reward Positivity (RewP) and Feedback‐Related Negativity (FRN) are ERP components that are sensitive to feedback valence, with FRN amplitudes typically being more negative following loss or negative feedback, while RewP amplitudes are more positive following gain or reward feedback (Foti et al., 2011; Pfabigan et al., 2014). Both components have been associated with increased ventral striatum and medial prefrontal cortex brain activity (Carlson et al., 2011). Neurophysiological deviations in reward delivery are somewhat better investigated in irritability or AD. Preschoolers diagnosed with DMDD were assessed later in preadolescence during a monetary reward task. This study found an association between DMDD symptoms assessed at age 3 and enhanced RewP to monetary rewards (Kessel et al., 2016), consistent with previous work on irritability in middle‐late childhood (Dougherty et al., 2015). In young adults, higher irritability was associated with reduced response to loss feedback, but across the whole sample, trait irritability was unrelated to reward responsivity (Deveney, 2019). An association between CD and reward responsivity was found to be moderated by irritability, such that youth with high CD symptom had less positive reward responsivity to win trials and more positive responsivity in loss trials, but this pattern was only observed in youth with high irritability scores (Waxmonsky et al., 2022). Taken together, findings on how reward delivery in irritability and AD is altered are mixed, showing both enhanced and reduced responses across studies. Therefore, we examined RewP and FRN amplitudes in this large sample of children with AD compared to peers without AD, without a directional hypothesis, to better clarify the direction and nature of these effects. Importantly, none of the above mentioned studies was based on a case‐control comparison and only the study of Waxmonsky et al., 2022 included a clinical sample, highlighting the need to follow this up in a larger cohort comparing those ERPs between AD and No‐AD peers.

Our study aims to increase our understanding of the relationship between reward processing and AD considering both the anticipatory CNV and the delivery RewP/FRN. We hypothesize that AD will show reduced anticipatory CNV and RewP/FRN activity, compared to a control group, reflecting altered cognitive anticipation and reward processing. Additionally, by adopting a dimensional approach that emphasizes measurable and continuous neurophysiological markers, we aim to assess how variations in reward‐related activity may explain AD symptoms, and by identifying the underlying neural mechanisms, we may enhance diagnostics and deepen our comprehension of the pathophysiology of AD. This potentially offers enhanced diagnostic precision and more targeted therapeutic interventions and increases our understanding of the phenotypic representation and the associated pathophysiology related to the conceptualization of AD.

## METHODS

### Study design and participants

Participants were part of the randomized, controlled trial ADOPT (AD—Optimizing Prevention and Treatment) (Döpfner et al., 2019). Participants were first selected from the ADOPT Epidemiology cohort, a large community‐based sample of 9759 children aged 8–12 years recruited through residents' registration offices in four major German cities (Treier et al., 2022). To define the high AD group, we used DADYS‐PQ scores falling within the top 10% of the distribution in this normative sample, consistent with established cut‐offs reflecting clinically relevant AD. The No‐AD group was similarly defined as children with DADYS‐PQ scores in the bottom 10% of the distribution. This approach allowed us to identify children at the extreme ends of the AD symptom continuum, in line with epidemiological prevalence estimates up to 9%, and prior validation of the DADYS‐Screen. Families of all children identified with AD, along with a randomly selected sample of families of children without AD, were invited to participate in a comprehensive assessment at one of five study centers. All families with children meeting the additional inclusion criteria were invited to take part in the subsequent treatment study, where they received either an AD‐specific treatment or standard care. Meanwhile, families without AD were monitored as a comparison group (ADOPT study, Döpfner et al., 2019). Here we report the data of the subgroup that participated in the ADOPT‐Neurobiology study, which was performed at the Technische Universität Dresden and the Central Institute of Mental Health, Mannheim. A total of 138 children (aged 8−12) participated in this EEG assessment. The full assessment battery consisted of three tasks: Emotional Stroop (Giller et al., 2021), Reward Anticipation (Kirsch et al., 2003) and Affective Posner Cueing Task (Rich et al., 2007) in addition to two 3 min resting state measurements (eyes open and eyes closed). Ethical approval for the study was obtained from the local ethics committee. Written consent/assent was obtained from the children and their legal guardians.

Children with an IQ of less than 80, with current or past traumatic brain injuries, with neurological diseases and/or with a confirmed diagnosis of bipolar disorder, manic episode, severe depression, or autism spectrum disorder were not included in the study. Moreover, AD should not be better explained by other disorders such as obsessive‐compulsive disorder, posttraumatic stress disorder, persistent depressive disorder, or substance use disorders. This exclusion criterion was evaluated by trained clinicians using the semi‐structured parent interview (DISYPS‐III; Döpfner & Görtz‐Dorten, 2017) to determine whether the symptom pattern was primarily attributable to AD or to another psychiatric condition. Assignment in the clinical participant group was based on screening scores in the top 10%, followed by confirmation by a certified clinician informed by the Diagnostic Tool for AD in Children (DADYS, Treier et al., 2023; Junghänel et al., 2022) as evaluated by a clinician based on a parent interview. Comorbidities such as ADHD, ODD, or CD or stimulant medication (e.g., methylphenidate) were not a reason for exclusion and are shown in Table 1. Children who were currently undergoing or planned to undergo intensive behavioral therapy were not included in the study. All children had a vision that was normal or corrected to normal.

**TABLE 1:** Sample characteristics.

	AD	No‐AD	T statistic	*p*
	(*N* = 50)	(*N* = 53)		
Age				
Mean (SD)	10.2 (1.44)	10.3 (1.51)	0.040	0.842
Median [min, max]	11.0 [7.00, 13.0]	10.0 [8.00, 13.0]		
IQ				
Mean (SD)	108 (13.1)	111 (12.2)	1.209	0.274
Median [min, max]	107 [83.0, 137]	112 [78.0, 134]		
Sex			*x* ^2^	
Female	15 (30.0%)	30 (56.6%)	7.402	0.006
Male	35 (70.0%)	23 (43.4%)		
Medication			*x* ^2^	
Yes	11 (22.0%)	1 (1.9%)	9.898	0.002
DADYS parent total scale				
Mean (SD)	1.30 (0.367)	0.168 (0.170)	407.718	<0.001
Median [min, max]	1.27 [0.538, 2.00]	0.154 [0, 0.769]		
CBCL depression/anxiety				
Mean (SD)	0.486 (0.351)	0.146 (0.166)	39.009	<0.001
Median [min, max]	0.462 [0, 1.54]	0.0769 [0, 0.615]		
CBCL aggression				
Mean (SD)	0.807 (0.316)	0.0780 (0.137)	229.252	<0.001
Median [min, max]	0.778 [0.111, 1.50]	0.0278 [0, 0.833]		
SCL ADHS				
Mean (SD)	1.15 (0.666)	0.293 (0.318)	68.032	<0.001
Median [min, max]	1.00 [0.100, 2.85]	0.200 [0, 1.40]		
ODD^a^				
Yes	17 (34.0%)			
DMDD^a^				
Yes	11 (22.0%)			
CD				
Yes	1 (2.0%)			
ADHD, combined type				
Yes	7 (14.0%)			
ADHD, predominantly inattentive type				
Yes	6 (12.0%)			
ADHD, predominantly hyperactive‐impulsive type				
Yes	1 (2.0%)			

Abbreviations: CBCL, Normalized child behavior check list subscale; DADYS, Diagnostic‐System for Affective Dysregulation; SCL, German Symptom Checklist for Attention‐deficit/hyperactivity disorder.

^a^ Although not possible in the DSM‐5 due to hierarchical rules, simultaneous DMDD and ODD diagnoses are reported here if all diagnostic criteria were met, due to interest in the diagnostic overlap.

### Reward anticipation task

Adapted from previous versions of the MID task (Kirsch et al., 2003), the paradigm was designed to measure reward anticipation and delivery of a monetary reward, and has been reported to activate the ventral striatum reliably and robustly (Boecker et al., 2014; Plichta et al., 2013). The visual information of the paradigm was presented on a monitor using Presentation software (Neurobehavioral Systems). As depicted in the Figure S1, the task requires the participant to respond by a fast button press once presented with a flash target. A fast press secures the target‐indicated reward. The smiley target cue indicates a potential monetary reward (0.50 Euros) and the scrambled smiley cue, a verbal feedback (i.e., “Fast reaction!”), which served as the control condition. The scrambled smiley with verbal feedback served as a non‐monetary control to isolate anticipatory and feedback processes specific to reward salience while controlling for visual, attentional, and motor components of task performance. This task did not include an explicit lose condition, only absence of reward (No Win: 0€ or “Unfortunately too slowly”). To encourage further engagement with the task, boost monetary trials of 2 Euros were included circa every eighth win trial. Participants were reminded of their overall account balance after every trial. The paradigm presented a total of 50 trials for each condition in a pseudo‐randomized order. Jittering of the cue duration was between 3 and 5s. The allocated reaction time window for a successful trial was adapted to account for between‐subject differences (max. 1 s), intended to support a comparable number of successful trials between participants (60% of trials). This time window remained constant between both conditions. Following measurement sessions, participants were paid their final earned balances (for details, see Figure S1).

## ASSESSMENT OF DEMOGRAPHIC INFORMATION AND CLINICAL CHARACTERIZATION

Demographic information was assessed before the EEG assessment. IQ was measured via the standardized short‐form of the German WISC IV (Petermann & Petermann, 2007), consisting of 4 subtests, namely Symbol Search and Vocabulary along with Matrix Reasoning and Block Design to confirm the inclusion criteria.

### DADYS questionnaire

The DADYS (Diagnostikum für Affektive Dysregulation [Diagnostic‐System for AD], Görtz‐Dorten & Döpfner, 2021), is a newly developed diagnostic system for the multimodal assessment of AD in children. Our analysis is based on the validated parent questionnaire (DADYS‐PQ, 36 items, Junghänel, Thöne, et al., 2022). This questionnaire contains *five domains of* core AD symptoms: functional impairment, irritability/emotional impulsivity, anger/irritability, positive emotionality and exuberance. Items of all DADYS instruments are rated on a 4‐point Likert scale (0 = not present to 3 = very strong).

### CBCL questionnaire

Using the Child Behavior Checklist 6/18 (CBCL; Achenbach, 2001), parents were able to report on the problems with behavior and emotions of their child. This analysis used the scales from this questionnaire, which measure anxiety/depression, attention difficulties and aggressive behavior (Döpfner et al., 2014).

### SCL‐ADHD questionnaire

Parent reports of ADHD symptoms were collected via the SCL‐ADHS form of the German Symptom Checklist for ADHD from the German diagnostic system for mental disorders in children and adolescents based on the ICD−10 and DSM‐5 (DISYPS‐III, Döpner & Görtz‐Dorten, 2017). The questionnaire comprised 20 items, which are rated on a four‐point likert scale ranging from 0 (not at all) to 3 (very much).

### EEG data acquisition and preprocessing

Participants were seated in a comfortable chair and fitted with a 60‐channel EEG cap (Brain Products Inc.). The EEG was recorded with a sampling rate of 500 Hz using a 72‐channel BrainAmp amplifier (Brain Products Inc.) and BrainVision Recorder software (Brain Products Inc.). An equidistant 60 Ag–AgCl‐EEG setup was used with the ground electrode at *θ* = 58, *φ* = 78 (between AFz and AF2) and the recording reference electrode Cz. Acquisition started after impedances for all channels were reduced to below 50 kΩ following standard data collection procedures (Ferree et al., 2001; Kappenman & Luck, 2010). One electrode was placed below the outer canthus of each eye (horizontal electrooculogram HEOG I & II) and one below the left eye (vertical electrooculogram VEOG I).

Offline processing was performed with Brain Vision Analyzer 2.2.0 (BrainProducts, Gilching Germany). The continuous EEG was filtered with 0.01–30 Hz, 24 db/oct Butterworth filters, broad artifacts were eliminated after visual inspection and heavily affected channels were interpolated using the spline type. Ocular artifacts were removed by an independent component analysis (ICA, infomax algorithm). Data were re‐referenced to the average and subsequently checked for remaining artifacts. Automated artifact rejection was applied for all segments using amplitude differences above 200 μV in a 200 ms time interval as well as activity ±150 μV as rejection criteria. For the anticipation phase, the CNV was the primary ERP measured from 2000 to 3000 ms window following cue onset at Cz and fronto‐central site (FCz), which commonly show the highest (more negative) amplitude in this task (Boecker et al., 2014; Plichta et al., 2013) and are typically more frontal at younger ages. To examine the feedback‐locked ERP responses, we analyzed both the RewP and the FRN, which, closely related and temporally overlapping, are commonly interpreted as indexing slightly distinct neural responses to outcome valence: RewP to reward receipt and FRN to loss or non‐reward (Foti et al., 2011; Hennefield et al., 2022; Zubovics et al., 2021). Based on the visual inspection of scalp grand‐averaged topographic plots and ERP waveforms, the RewP was scored as the mean win‐no‐win difference amplitude from 175 to 275 ms following feedback onset, consistent with prior work (e.g., Zubovics et al., 2021), and the FRN as the mean win‐no‐win difference from 250 to 350 ms. Mean amplitude was extracted for selected electrodes (RewP: AFz, AF3, Fz, and AF4; FRN: FCz, Cz CPz, CP1 and CP2). The difference in activity between win and no win was calculated per participant for *each condition* (verbal and monetary) before group‐level averaging.

### Statistical analysis

Sample characteristics were analyzed using analysis of variance (ANOVA) and chi‐square tests, when appropriate. Behavioral data (Reaction time) were analyzed using repeated measures ANOVA, with one within‐factor for condition (monetary and verbal) and one between‐factor for group (AD and No‐AD). ERP reward anticipation data were analyzed using repeated measures ANOVA, with two within‐factors for condition (monetary and verbal) and electrodes (Cz and FCz) and one between‐factor for group (AD and No‐AD). EEG reward delivery data (RewP and FRN separately) were analyzed using repeated measures ANOVA, with one within‐factor for condition (monetary and verbal) and one between‐factor for group (AD and No‐AD). All analyses were adjusted for sex as a covariate of non‐interest. Additionally, a sensitivity analysis was performed, including site and medication as covariates, and all analyses were repeated using only participants with available behavioral data. Cohen's *d* and partial *η* ^2^ effect sizes were reported (Cohen, 1988).

To determine the extent to which EEG reward‐related activity can explain AD symptoms, as measured by the continuous DADYS‐PQ total score, two stepwise linear regression models were applied including both groups. The first regression assessed how much variance can be explained by EEG activity, with sex and medication added as control variables in the first step. In the second step, EEG activity for reward anticipation at Cz and FCz was added, and in the third step, the RewP for the reward delivery phase were introduced. The same model was repeated, adding continuous measures of ADHD (SCL‐ADHD score), aggression and anxiety/depressive (CBCL scores) symptoms together as a second step. In the third step, reward anticipation at Cz and FCz was included, and in the fourth step, the RewP or FRN for the reward delivery phase was introduced.

To complement the primary regression analyses focused on predicting AD symptoms from EEG measures, a second set of regression models was conducted to explore which symptom dimensions—including core features of AD, measured by DADYS‐PQ—were uniquely associated with the reward‐related ERP components. These models were also controlled for sex and medication, with the dependent variables being CNV reward anticipation activity at FCz, RewP or FRN, and the independent variables being key symptoms of AD or comorbidities, such as aggression, anxiety/depression, and ADHD. Correction for multiple testing was performed for these regression analyses applying a Bonferroni correction for 8 tests (0.05/8 = 0.00625). All analysis were performed with SPSS, Version 26 and *R* Studio v4.1.2.

## RESULTS

### Sample characteristics

Table 1 shows the sample characteristics. From the 138 participants available for EEG analysis, 35 participants could not be included in the final analysis because of errors in recordings or poor quality of the EEG data (less than 15 segments/epochs each condition for RewP/FRN and 20 segments/epochs each condition of the anticipation phase. For full details, see Tables S1 and S2). For analysis of the reward anticipation phase, 103 participants (53 No‐AD and 50 AD) were included. Compared to No‐AD, the AD group consisted of more males (*χ* ^2^ = 7.402, *p* < 0.001). For the reward delivery phase, slightly fewer participants (RewP, *n* = 96, No‐AD = 52, AD = 44; FRN, *n* = 101, No‐AD = 53, AD = 48) could be analyzed due to higher presence of movement artifacts and the presence of one outlier. For the reaction time data 93 participants could be analyzed (No‐AD = 52, AD = 41) due to further technical problems. Furthermore, no significant differences were found for the baseline characteristics and symptom severity of the excluded participant's versus the included ones.

### Behavioral data

Reaction Times were faster following monetary cues [*F* (1, 90) = 7.012, *p* = 0.010, part. *η*2 = 0.072]. Both groups performed similarly and no significant interaction and group effect was found (all *p* > 0.489). For details, see Figure S2.

### Anticipation

A significant group × condition interaction [*F* (1,100) = 5.409, *p* = 0.022, part. *η* ^2^ = 0.051] revealed that the AD group had reduced anticipatory CNV amplitude. Post‐hoc test showed that this between‐group difference was stronger for the cue monetary condition (monetary cue: *p* = 0.007, *d* = 0.56, verbal cue: *p* = 0.901, *d* = 0.16), and that only the No‐AD group showed a significant difference between conditions (*p* < 0.001). A significant group × channel interaction [*F* (1,100) = 4.052, *p* = 0.047, part. *η* ^2^ = 0.039] revealed that the group differences were more pronounced at the fronto‐central electrode (FCz, *p* = 0.017). No other significant effects or interactions emerged.

**FIGURE 1:**
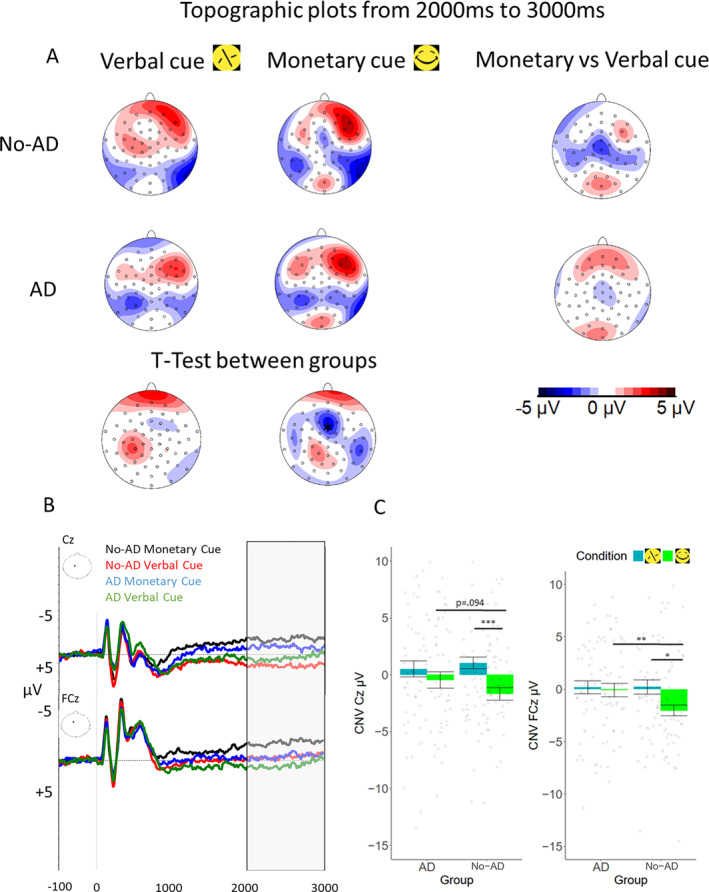
Reward anticipation CNV 2000—3000 ms. (A) Topographic plots from 2000 to 3000 ms. (B) ERPs at Cz and fronto‐central site (FCz). (C) Extracted mean activity at Cz and FCz for both groups and conditions. No‐AD, Participants without affective dysregulation, AD, Affective dysregulation. ***p < 0.001, **p < 0.01, *p < 0.05.

For details see Figure 1 and Table 2.

**TABLE 2:** Descriptive statistics of ERPs.

	AD	No‐AD	Cohen's *d*
	(*N* = 50)	(*N* = 53)	
Monetary cue CNV (FCz)			
Mean (SD)	0.469 (5.13)	−2.02 (3.78)	0.56**
Median [min, max]	−0.404 [−8.83, 16.3]	−2.32 [−13.6, 7.41]	95% CI [0.16, 0.95]
Verbal cue CNV (FCz)			
Mean (SD)	0.784 (5.11)	−0.0770 (5.35)	0.16
Median [min, max]	0.489 [−11.3, 18.4]	−0.444 [−16.1, 9.88]	95% CI [−0.22, 0.55]
Monetary cue CNV (Cz)			
Mean (SD)	−0.233 (5.22)	−1.98 (4.53)	0.36
Median [min, max]	−0.665 [−12.0, 10.8]	−1.68 [−17.9, 6.55]	95% CI [−0.03, 0.75]
Verbal cue CNV (Cz)			
Mean (SD)	0.513 (4.97)	1.23 (3.91)	−0.16
Median [min, max]	1.10 [−13.5, 9.15]	0.166 [−5.15, 11.0]	95% CI [−0.55, 0.23]
Verbal RewP			
Mean (SD)	1.22 (3.86)	1.73 (5.33)	−0.14
Median [min, max]	0.830 [−5.56, 12.6]	1.83 [−11.8, 15.0]	95% CI [−0.51, 0.29]
Missing	6 (12.0%)	1 (1.9%)	
Monetary RewP			
Mean (SD)	−0.214 (3.50)	1.65 (4.59)	−0.45°
Median [min, max]	0.0465 [−9.08, 8.02]	0.897 [−7.02, 15.4]	95% CI [−0.86, −0.05]
Missing	6 (12.0%)	1 (1.9%)	
Verbal FRN			
Mean (SD)	1.44 (3.12)	0.673 (2.89)	0.25
Median [min, max]	1.27 [−4.28, 7.97]	0.542 [−4.59, 7.16]	95% CI [−0.14, 0.65]
Missing	2 (4.0%)	0 (0%)	
Monetary FRN			
Mean (SD)	0.0715 (3.18)	0.300 (2.71)	−0.08
Median [min, max]	−0.336 [−7.59, 6.19]	0.112 [−5.36, 5.73]	95% CI [−0.47, 0.31]
Missing	2 (4.0%)	0 (0%)	

Abbreviations: AD, Affective dysregulation; CNV, Contingent negative variation; FRN, Feedback related negativity; RewP, Reward positivity; SD, Standard deviation.

**p < 0.01; °*p* < 0.1.

### Delivery

**FIGURE 2:**
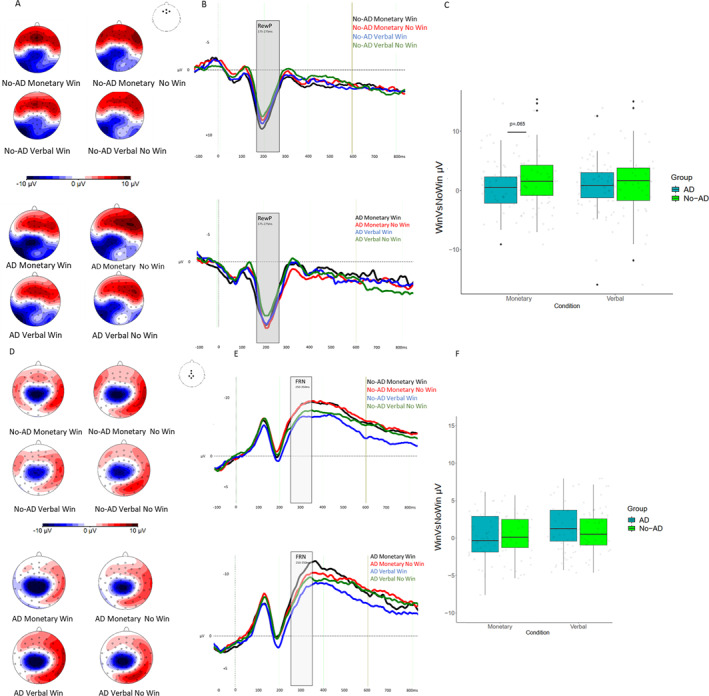
Reward delivery reward positivity (RewP) 175–275 ms. (A) Displays the topographies for No‐AD (Top) and Affective dysregulation (AD) (bottom) for each condition. (B) Displays the ERPs for No‐AD (Top) and AD (bottom) for each condition. (C) Extracted difference between Win and No win for each condition by group. Feedback‐Related Negativity (FRN) 250—350 ms. (D) Displays the topographies for No‐AD (Top) and AD (bottom) for each condition. (E) Displays the ERPs for No‐AD (Top) and AD (bottom) for each condition. (F) Extracted difference between Win and No win for each condition by group. AD, Affective dysregulation.

No significant main effect or interaction emerged neither for RewP or FRN, but the AD group tended to show a less pronounced RewP at the selected fronto‐central electrodes for the monetary condition (*p* = 0.065, *d* = −0.45). For details see Figure 2 and Table 2.

### Sensitivity analysis

We additionally performed a sensitivity analysis, including medication and site as a separate covariate and using only participants with available behavioral data, which all did not impact the above mentioned results. Furthermore, age was examined in exploratory correlations and showed no significant association with CNV, RewP, or FRN amplitudes (all *p* > 0.128); therefore, it was not retained as a covariate. For more details see Appendix S1 and Table S3.

### Regression analysis

**FIGURE 3:**
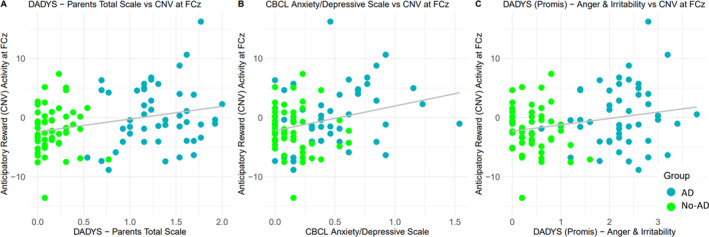
Scatter plot—regression analyses. (A) Scatter plot showing a positive association between Affective dysregulation (AD) core symptoms and the anticipatory reward (CNV) activity at fronto‐central site (FCz) (*b* = 0.251, *p* = 0.0105). (B) Showing a positive association between the anticipatory reward (CNV) activity at FCz and Anxiety/Depression symptoms (*b* = 0.301, *p* = 0.003) measured via the CBCL (normalized). (C) Showing a positive association between the anticipatory reward (CNV) activity at FCz and Anger and Irritability symptoms (*b* = 0.299, *p* = 0.004). AD, Affective dysregulation.

In the first step, including only sex and medication to predict AD symptoms, the model was significant [*R* ^2^ = 0.116, *F* (2, 92) = 6.029, *p* = 0.003], explaining 11% of the variance. Only medication was a significant predictor of AD symptoms (*b* = 0.251, *p* = 0.0105). In the second step, incorporating also the anticipatory reward CNV from FCz and Cz, resulted in a 7.8% improvement in the model [*R* ^2^ change = 0.078, *F* (2, 90) = 4,375, *p* = 0.015]. Only the CNV activity for the monetary condition at FCz was a significant predictor of AD symptoms (*b* = 0.283, *t* = 2.728 *p* = 0.008, Figure 3 A). In the final step, we included the delivery phase ERP activity, which did not significantly improve the model (*p* = 0.161). When repeating the regression analysis, but including ADHD, aggression and anxiety/depression symptoms, in a second step, 79% of the variance was explained. This high proportion was expected, since these symptoms share considerable variance with AD and the selection criteria for our AD and No‐AD group. The variance inflation factors were below 2.5, indicating that multicollinearity was not problematic. Including the anticipatory reward CNV from FCz and Cz increased the model significantly by 1.7% (*R* ^2^ change = 0.017, *p* = 0.023). The delivery phase ERPs yielded no significant model improvement (*p* > 0.525). For details, see Tables S4 and S5.

In a further stepwise regression model, we examined the dimensions related to the significant diminished reward anticipation activity at FCz. We observed that anxiety and depressive symptoms (*b* = 0.301, *t* = 3.049, *p* = 0.003, Figure 3B) and anger and irritability (*b* = 0.299, *t* = 2.947, *p* = 0.004, Figure 3C), accounting for 10% and 9.3% of the variance, were significantly associated with FCz activity. These associations also withstood correction for multiple testing. Regarding RewP and FRN, we found a significant negative association also with anxiety and depressive symptoms (*b* = −0.259, *t* = −2.581, *p* = 0.011) and RewP, although this did not withstand correction for multiple testing and should be interpreted as explorative. For details, see Table S6.

## DISCUSSION

The current study examined differences in anticipatory and delivery‐related reward processing between children with and without AD, and investigated their associations with well‐characterized AD symptoms. This provides a deeper understanding of the conceptualization of AD and its neurobiological mechanisms.

Concerning reward anticipation processes, this study revealed a reduced anticipatory CNV amplitude in AD, specifically at fronto‐central electrodes. The reduction was most noticeable in the monetary cue condition, indicating a compromised or less active anticipatory reward process in AD children, suggesting compromised processing in the positive valence domain of the RDoC framework. Deficiencies in reward anticipation potentially reflect a blunted attribution of expectancy and motivational salience (Hägele et al., 2015) for rewarding stimuli which might contribute to difficulties in reward learning processes (Adleman et al., 2011). Furthermore, as previously shown, children with AD showed impaired ability to process conflicting emotional information (Giller et al., 2022). This impairment might extend to the processing of reward‐related cues resulting in difficulties selecting an appropriate response and partially mediating also frustrative non‐reward responses (Brotman et al., 2017), and contribute to unrealistic expectations, which might cause temper outburst when the expected reward fails to materialize (Wakschlag et al., 2012).

Moreover, our reward‐delivery findings revealed no significant differences in RewP or FRN between the AD and No‐AD. The absence of significant effects may reflect the considerable heterogeneity of findings across studies and designs investigating the RewP or FRN (i.e., Hennefield et al., 2022; Proudfit, 2015; Zubovics et al., 2021). Nonetheless, the trend‐level difference indicating slightly less pronounced RewP in the AD group for the monetary condition should be investigated in future larger studies, as a reduced RewP might also imply impaired reward learning (Adleman et al., 2011) and reward sensitivity (Perlman et al., 2015) consistent with the frustrative non‐reward hypothesis.

In line with a dimensional perspective (i.e., Hierarchical Taxonomy of Psychopathology and RDoC), classified as a high‐priority research goal (Leibenluft et al., 2024), we performed several regression analyses. First, we showed that the CNV reward anticipation activity (reduced in AD) explained an additional 7.8% of the variance in AD after including medication and sex but only an additional 1.7% after including core AD symptoms, informing the limited additional diagnostic utility of the EEG assessment. However, anger/irritability and anxiety and depressive symptoms predicted a reduced CNV reward anticipation activity at FCz. This suggests that these symptoms contribute to deficits in reward expectation and anticipation, being in line with the studies linking reduced anticipatory activity with anhedonia and depressive symptoms (Ait Oumeziane et al., 2019; Stringaris et al., 2015). Furthermore, the prevailing consensus suggests that youth irritability predicts internalizing disorders in later life, particularly depression (for a meta‐analysis, see Vidal‐Ribas et al., 2016). It has also been proposed that irritability is a mood manifestation that shares common risk factors with depressive and anxiety disorders (Stringaris & Taylor, 2015). Interestingly, in our study, only reward anticipation demonstrated a robust link with internalizing symptoms. In contrast to prior research findings (i.e., Belden et al., 2016; Bress et al., 2013; Burkhouse et al., 2017), the RewP showed a weaker association. Our results highlight that anger/irritability and depressive and anxiety symptoms are linked to blunted reward anticipation and expectation. Since AD is a highly transdiagnostic concept associated with both externalizing and internalizing symptoms (Leibenluft et al., 2024), deficits in reward processing at a neural level might point to an important contribution of internalizing problems. This may reflect tonic irritability which is characterized by a persistently angry or grumpy mood and linked to internalizing psychopathology and suicidal behavior (Sorcher et al., 2024). This insight may not only enable us to improve and tailor diagnostics and develop more targeted therapies, such as by systematically increasing engagement in rewarding social and interpersonal activities (Solomonov, 2023) and/or with a treatment targeting reward anticipation and motivation through pleasurable activities and imagining positive future outcomes (Craske et al., 2023), it might also suggest that a diminished neural response to anticipated reward could serve as an early indicator for developing depressive and anxiety disorders in later life and possibly inform early prevention and personalized treatment strategies.

However, this research is not without limitations. Our design, which dichotomized participants into the top and bottom 10% of AD scores, excluded children with moderate levels of dysregulation, potentially limiting the generalizability of the findings across the full symptom continuum. In addition, the higher proportion of males in the AD group may reflect a gender‐related reporting bias, whereby irritability and aggression are more readily recognized in boys, which warrants further investigation. The RewP and FRN are commonly used in an exchangeable manner within a heterogeneous time‐range (i.e., Hennefield et al., 2022; Proudfit, 2015; Zubovics et al., 2021), which we addressed by covering both and specifically replicating the time‐range of Zubovics et al., 2021. Furthermore, the reward anticipation task used was not designed to test frustration per se. The link to frustrative nonreward should be followed up with specific frustration tasks in the future, such as the affective Posner cueing task. Additionally, the absence of significant findings for the RewP/FRN might be attributable to the reduced number of available trials (in the present study, a minimum of 15 trials). Although we did not calculate internal consistency within this dataset, prior work indicates that RewP/FRN estimates based on eight or more trials can show acceptable reliability (Ethridge & Weinberg, 2018; Paul et al., 2025). Furthermore, information on race and ethnicity was not systematically collected, which limits the assessment of demographic generalizability. Lastly, longitudinal data would be needed to clarify how the relation between reward anticipation, AD and internalizing symptom evolves over time.

## CONCLUSION

In conclusion, our findings contribute to the body of evidence on altered reward processing in AD by implicating deviant neural activity in anticipation of reward preceding processes related to the delivery of the reward itself. Furthermore, our results underscore the importance of dimensional analyses which showed that both anger/irritability and anxiety/depressive symptoms are relevant dimensions in the pathophysiology of AD for atypical reward anticipation. This understanding offers promising directions for future research and might improve diagnostics and future interventions.

## AUTHOR CONTRIBUTIONS

**Pascal‐M. Aggensteiner**: Writing—original draft; preparation (lead); formal analysis (lead). **Francisca Giller**: Writing—review and editing (equal). **Nathalie Holz**: Writing—review and editing (equal). **Anna Kaiser:** Writing—review and editing (equal). **Christine Igel**: Writing—review and editing (equal). **Sarah Hohmann**: Conceptualization (supporting); writing—original draft (supporting); writing—review and editing (equal); funding acquisition. **Veit Roessner**: Conceptualization (supporting); writing—original draft (supporting); writing—review and editing (equal); funding acquisition. **Christian Beste**: Conceptualization (supporting); writing—original draft (supporting); writing—review and editing (equal); funding acquisition. **Tobias Banaschewski**: Conceptualization (supporting); writing—original draft (supporting); writing—review and editing (equal); funding acquisition. **Daniel Brandeis**: Conceptualization (supporting); writing—original draft (supporting); writing—review and editing (equal); funding acquisition. All authors critically revised the manuscript for important intellectual content, and all authors gave final approval of the latest version of the manuscript and agreed to be accountable for all aspects of the work in ensuring that questions related to the accuracy or integrity of any part of the work are appropriately investigated and resolved.

## CONFLICT OF INTEREST STATEMENT

Tobias Banaschewski served in an advisory or consultancy role for eye level, Infectopharm, Lundbeck, Medice, Neurim Pharmaceuticals, Oberberg GmbH, Roche, and Takeda. He received conference support or speaker's fee by Janssen, Medice and Takeda. He received royalties from Hogrefe, Kohlhammer, CIP Medien, and Oxford University Press. M.D. receives royalties from publishing companies as an author of books and treatment manuals on child behavioral therapy and of assessment manuals published by Beltz, Elsevier, Enke, Guilford, Hogrefe, Huber, Kohlhammer, Schattauer, Springer, and Wiley. He receives income as a consultant for Child Behavior Therapy at the National Association of Statutory Health Insurance Physicians. He also receives consulting income and research support from Lilly, Medice, Takeda, and eyelevel GmbH. Sarah Hohmann has received speakers fees from Infectopharm. Veit Roessner has received lecture honoraria from Infectopharm and Medice companies. He has carried out clinical trials in cooperation with Servier and Shire Pharmaceuticals/Takeda companies. Daniel Brandeis served as an unpaid scientific consultant for an EU‐funded neurofeedback trial. The present work is unrelated to the above grants and relationships. The remaining authors have declared that they have no competing or potential conflicts of interest.

## ETHICAL CONSIDERATIONS

Ethical approval for the study was obtained from the local ethics committee of the Technische Universität Dresden (EK495122012) and the Central Institute of Mental Health, Mannheim (2018−546N‐MA, 03.04.2018). Written consent/assent was obtained from the children and their legal guardians.

## Supporting information

Supporting Information S1

## Data Availability

Data are available upon reasonable request after the publication of the main results of the ADOPT study.
