# Supplementary material for: Reward processing in children with affective dysregulation
Source: JCPP Adv. 2026 Mar 7:e70102. Online ahead of print. doi: 10.1002/jcv2.70102 (PMC13339600; doi:10.1002/jcv2.70102)
Supplement: Supplementary file 1 — Supporting Information S1 [file JCV2-9999-e70102-s001.docx]

**Reward processing in children with Affective Dysregulation**

**Supporting Information**

Table S1 - Interpolated channels

| Interpolated channels | |  |  |
| --- | --- | --- | --- |
| Mean | median | min | max |
| 2,77 | 2 | 0 | 15 |

| Channel | N | Channel | N | Channel | N | Channel | N |
| --- | --- | --- | --- | --- | --- | --- | --- |
| P11 | 20 | P12 | 9 | **AF3** | **5** | P8 | 3 |
| Fp2 | 18 | O9 | 8 | CP1 | 4 | **AFZ** | **2** |
| Fp1 | 14 | O1 | 7 | FT7 | 4 | C3 | 2 |
| **Fpz** | **13** | Iz | 6 | T8 | 4 | P9 | 2 |
| FP2 | 12 | TP9 | 6 | AF4 | 4 | P10 | 2 |
| FP1 | 12 | O10 | 5 | IZ | 4 | P1 | 2 |
| AF8 | 11 | Oz | 5 | O2 | 3 | FC2 | 2 |
| FT10 | 11 | TP10 | 5 | F5 | 3 | CP6 | 2 |
| AF7 | 10 | TP7 | 5 | FT8 | 3 | CZ | 2 |
| FT9 | 10 | TP8 | 5 | T7 | 3 | FC1 | 2 |
|  |  |  |  |  |  |  |  |
|  |  |  |  |  |  |  |  |
| Channel | N | Channel | N |  |  |  |  |
| F6 | 2 | PO2 | 1 |  |  |  |  |
| F2 | 2 | C4 | 1 |  |  |  |  |
| FZ | 2 | PO1 | 1 |  |  |  |  |
| C5 | 1 | CPz | 1 |  |  |  |  |
| PZ | 1 | CP2 | 1 |  |  |  |  |
| FC6 | 1 | P7 | 1 |  |  |  |  |
| OZ | 1 | P4 | 1 |  |  |  |  |
| FC5 | 1 | P3 | 1 |  |  |  |  |
| CP4 | 1 | CP3 | 1 |  |  |  |  |
| CP5 | 1 |  |  |  |  |  |  |
| Pz | 1 |  |  |  |  |  |  |

In Bold channels used for analysis

Table S2a – Segments/Epochs – Reward Anticipation

| **AD** |  |  |  |  |  |
| --- | --- | --- | --- | --- | --- |
| **Descriptive statistics** | | | | | |
|  | N | Minimum | Maximum | Mean | Std |
| Cz_Monetary cue | 50 | 20 | 50 | 47,54 | 5,11 |
| FCz_Monetary cue | 50 | 20 | 50 | 48,65 | 2,42 |
| Cz_Verbal_cue | 50 | 20 | 50 | 47,65 | 5,39 |
| FCz_Verbal_cue | 50 | 20 | 50 | 48,54 | 4,17 |
|  |  |  |  |  |  |
| **No-AD** |  |  |  |  |  |
| **Descriptive statistics** | | | | | |
|  | N | Minimum | Maximum | Mean | Std |
| Cz_Monetary cue | 53 | 20 | 50 | 47,46 | 5,42 |
| FCz_Monetary cue | 53 | 20 | 50 | 46,54 | 6,89 |
| Cz_Verbal_cue | 53 | 20 | 50 | 49,17 | 3,04 |
| FCz_Verbal_cue | 53 | 20 | 50 | 48,73 | 3,58 |

Table S2b – Segments/Epochs – Reward Delivery Monetary Win

***RewP***

| **AD** |  |  |  |  |  | **No-AD** |  |  |  |  |  |  |
| --- | --- | --- | --- | --- | --- | --- | --- | --- | --- | --- | --- | --- |
|  |  |  |  |  |  |  |  |  |  |  |  |  |
| **Descriptive statistics** | | | | | |  | **Descriptive statistics** | | | | | |
|  | N | Minimum | Maximum | Mean | Std |  |  | N | Minimum | Maximum | Mean | Std |
| AFz | 44 | 15 | 50 | 27,09 | 8,083 |  | AFz | 52 | 15 | 50 | 25,788 | 7,8324 |
| FCz | 44 | 15 | 50 | 27,21 | 8,222 |  | FCz | 52 | 15 | 50 | 25,79 | 7,865 |
| AF3 | 44 | 15 | 50 | 27,17 | 8,031 |  | AF3 | 52 | 15 | 50 | 25,596 | 7,8572 |
| AF4 | 44 | 15 | 50 | 27,11 | 8,141 |  | AF4 | 52 | 15 | 50 | 25,654 | 7,8263 |
| Fz | 44 | 15 | 50 | 27,19 | 8,155 |  | Fz | 52 | 15 | 50 | 25,81 | 7,849 |
|  |  |  |  |  |  |  |  |  |  |  |  |  |
|  |  |  |  |  |  |  |  |  |  |  |  |  |
|  |  |  |  |  |  |  |  |  |  |  |  |  |
| ***FRN*** |  |  |  |  |  |  |  |  |  |  |  |  |
|  |  |  |  |  |  |  |  |  |  |  |  |  |
|  |  |  |  |  |  |  |  |  |  |  |  |  |
| **AD** |  |  |  |  |  | **No-AD** |  |  |  |  |  |  |
|  |  |  |  |  |  |  |  |  |  |  |  |  |
| **Descriptive statistics** | | | | | |  | **Descriptive statistics** | | | | | |
|  | N | Minimum | Maximum | Mean | Std | |  | N | Minimum | Maximum | Mean | Std |
| FCz | 48 | 15 | 50 | 27,21 | 8,222 |  | FCz | 53 | 15 | 50 | 25,79 | 7,865 |
| Cz | 48 | 15 | 50 | 27,06 | 8,221 |  | Cz | 53 | 15 | 50 | 25,344 | 8,1791 |
| CPz | 48 | 15 | 50 | 27,23 | 8,220 |  | CPz | 53 | 15 | 50 | 25,84 | 7,855 |
| CP1 | 48 | 15 | 50 | 27,28 | 8,208 |  | CP1 | 53 | 15 | 50 | 25,904 | 7,7921 |
| CP2 | 48 | 15 | 50 | 27,23 | 8,204 |  | CP2 | 53 | 15 | 50 | 25,83 | 7,866 |
|  |  |  |  |  |  |  |  |  |  |  |  |  |

Table S2c – Segments/Epochs – Reward Delivery Monetary NoWin

***RewP***

| **AD** |  |  |  |  |  | **No-AD** | |  |  |  |  |  |
| --- | --- | --- | --- | --- | --- | --- | --- | --- | --- | --- | --- | --- |
|  |  |  |  |  |  |  |  |  |  |  |  |  |
|  |  |  |  |  |  |  |  |  |  |  |  |  |
| **Descriptive statistics** | | | | | |  | **Descriptive statistics** | | | | | |
|  | N | Minimum | Maximum | Mean | Std |  |  | N | Minimum | Maximum | Mean | Std |
| AFz | 44 | 15 | 50 | 25,326 | 7,8346 |  | AFz | 52 | 15 | 50 | 23,212 | 6,8294 |
| FCz | 44 | 15 | 50 | 25,52 | 7,808 |  | FCz | 52 | 15 | 50 | 23,29 | 6,804 |
| AF3 | 44 | 15 | 50 | 25,217 | 7,8029 |  | AF3 | 52 | 15 | 50 | 23,038 | 6,8369 |
| AF4 | 44 | 15 | 50 | 25,370 | 7,8609 |  | AF4 | 52 | 15 | 50 | 23,135 | 6,7679 |
| Fz | 44 | 15 | 50 | 25,543 | 7,7937 |  | Fz | 52 | 15 | 50 | 23,27 | 6,788 |
|  |  |  |  |  |  |  |  |  |  |  |  |  |
|  |  |  |  |  |  |  |  |  |  |  |  |  |
|  |  |  |  |  |  |  |  |  |  |  |  |  |
| **AD** |  | ***FRN*** |  |  |  | **No-AD** | |  |  |  |  |  |
|  |  |  |  |  |  |  |  |  |  |  |  |  |
| **Descriptive statistics** | | | | | |  | **Descriptive statistics** | | | | | |
|  | N | Minimum | Maximum | Mean | Std |  |  | N | Minimum | Maximum | Mean | Std |
| FCz | 48 | 15 | 50 | 25,52 | 7,808 |  | FCz | 53 | 15 | 50 | 23,29 | 6,804 |
| Cz | 48 | 15 | 50 | 25,478 | 7,8166 |  | Cz | 53 | 15 | 50 | 23,077 | 6,8795 |
| CPz | 48 | 15 | 50 | 25,565 | 7,7879 |  | CPz | 53 | 15 | 50 | 23,29 | 6,804 |
| CP1 | 48 | 15 | 50 | 25,457 | 7,7766 |  | CP1 | 53 | 15 | 50 | 23,288 | 6,8035 |
| CP2 | 48 | 15 | 50 | 25,435 | 7,8787 |  | CP2 | 53 | 15 | 50 | 23,27 | 6,809 |
|  |  |  |  |  |  |  |  |  |  |  |  |  |

Table S2d – Segments/Epochs – Reward Delivery Verbal Win

***RewP***

|  | **AD** |  |  |  |  | **No-AD** | |  |  |  |  |
| --- | --- | --- | --- | --- | --- | --- | --- | --- | --- | --- | --- |
| **Descriptive statistics** | | | | | | **Descriptive statistics** | | | | | |
|  | N | Minimum | Maximum | Mean | Std |  | N | Minimum | Maximum | Mean | Std |
| AFz | 44 | 15 | 50 | 29,23 | 11,807 | AFz | 52 | 15 | 50 | 30,000 | 12,6445 |
| FCz | 44 | 15 | 50 | 29,49 | 11,758 | FCz | 52 | 15 | 50 | 31,25 | 13,214 |
| AF3 | 44 | 15 | 50 | 29,02 | 11,884 | AF3 | 52 | 15 | 50 | 31,173 | 12,9868 |
| AF4 | 44 | 15 | 50 | 29,13 | 11,896 | AF4 | 52 | 15 | 50 | 30,923 | 12,8183 |
| Fz | 44 | 15 | 50 | 29,45 | 11,743 | Fz | 52 | 15 | 50 | 31,13 | 13,160 |
|  |  |  |  |  |  |  |  |  |  |  |  |
|  |  |  |  |  |  |  |  |  |  |  |  |
| ***FRN*** |  |  |  |  |  |  |  |  |  |  |  |
|  | **AD** |  |  |  |  | **No-AD** | |  |  |  |  |
| **Descriptive statistics** | | | | | | **Descriptive statistics** | | | | | |
|  | N | Minimum | Maximum | Mean | Std |  | N | Minimum | Maximum | Mean | Std |
| FCz | 48 | 15 | 50 | 29,49 | 11,758 | FCz | 53 | 15 | 50 | 31,25 | 13,214 |
| Cz | 48 | 15 | 50 | 29,62 | 11,793 | Cz | 53 | 15 | 50 | 31,442 | 13,6345 |
| CPz | 48 | 15 | 50 | 29,49 | 11,823 | CPz | 53 | 15 | 50 | 31,04 | 13,333 |
| CP1 | 48 | 15 | 50 | 29,51 | 11,834 | CP1 | 53 | 15 | 50 | 31,769 | 13,5670 |
| CP2 | 48 | 15 | 50 | 29,47 | 12,034 | CP2 | 53 | 15 | 50 | 31,52 | 13,283 |
|  |  |  |  |  |  |  |  |  |  |  |  |

Table S2e – Segments/Epochs – Reward Delivery Verbal NoWin

***RewP***

| **AD** |  |  |  |  |  |  | **No-AD** |  |  |  |  |  |
| --- | --- | --- | --- | --- | --- | --- | --- | --- | --- | --- | --- | --- |
|  |  |  |  |  |  |  |  |  |  |  |  |  |
| **Descriptive statistics** | | | | | |  | Descriptive statistics | |  |  |  |  |
|  | N | Minimum | Maximum | Mean | Std |  |  | N | Minimum | Maximum | Mean | Std |
| AFz | 44 | 15 | 50 | 27,500 | 10,3468 |  | AFz | 52 | 15 | 50 | 27,230 | 9,2690 |
| FCz | 44 | 15 | 50 | 27,52 | 10,407 |  | FCz | 52 | 15 | 50 | 27,27 | 9,489 |
| AF3 | 44 | 15 | 50 | 27,413 | 10,5527 |  | AF3 | 52 | 15 | 50 | 26,942 | 9,3817 |
| AF4 | 44 | 15 | 50 | 27,565 | 10,3573 |  | AF4 | 52 | 15 | 50 | 27,120 | 9,5250 |
| Fz | 44 | 15 | 50 | 27,54 | 10,436 |  | Fz | 52 | 15 | 50 | 27,37 | 9,470 |
|  |  |  |  |  |  |  |  |  |  |  |  |  |
| ***FRN*** |  |  |  |  |  |  |  |  |  |  |  |  |
|  |  |  |  |  |  |  |  |  |  |  |  |  |
| **AD** |  |  |  |  |  |  | **No-AD** |  |  |  |  |  |
|  |  |  |  |  |  |  |  |  |  |  |  |  |
| **Descriptive statistics** | | | | | |  | **Descriptive statistics** | | | | | |
|  | N | Minimum | Maximum | Mean | Std |  |  | N | Minimum | Maximum | Mean | Std |
| FCz | 48 | 15 | 50 | 27,52 | 10,407 |  | FCz | 53 | 15 | 50 | 27,27 | 9,489 |
| Cz | 48 | 15 | 50 | 27,457 | 10,2820 |  | Cz | 53 | 15 | 50 | 27,04 | 9,667 |
| CPz | 48 | 15 | 50 | 27,54 | 10,439 |  | CPz | 53 | 15 | 50 | 27,71 | 9,841 |
| CP1 | 48 | 15 | 50 | 27,630 | 10,4080 |  | CP1 | 53 | 15 | 50 | 27,346 | 9,4555 |
| CP2 | 48 | 15 | 50 | 27,70 | 10,469 |  | CP2 | 53 | 15 | 50 | 27,35 | 9,456 |
|  |  |  |  |  |  |  |  |  |  |  |  |  |

Table S3 – Correlation Matrix with Age
Pearson correlations among ERP measures, age, and reward-related indices in the full sample (N = 103)

| **Variable** | **1** | **2** | **3** | **4** | **5** | **6** | **7** | **8** | **9** |
| --- | --- | --- | --- | --- | --- | --- | --- | --- | --- |
| 1. Age | — | −.059 | −.008 | −.070 | −.067 | .065 | −.037 | −.149 | −.103 |
| 2. FRN (Verbal) | −.059 | — | .454** | .162 | −.005 | −.119 | −.027 | −.004 | −.063 |
| 3. FRN (Monetary) | −.008 | .454** | — | .064 | .129 | −.240* | −.158 | −.088 | −.173 |
| 4. RewP (Verbal) | −.070 | .162 | .064 | — | .297** | .052 | −.007 | −.116 | −.135 |
| 5. RewP (Monetary) | −.067 | −.005 | .129 | .297** | — | −.228* | −.029 | −.231* | −.067 |
| 6. Cz CNV CueWin | .065 | −.119 | −.240* | .052 | −.228* | — | .398** | .411** | .206* |
| 7. Cz CNV CueNoWin | −.037 | −.027 | −.158 | −.007 | −.029 | .398** | — | .131 | .501** |
| 8. FCz CNV CueWin | −.149 | −.004 | −.088 | −.116 | −.231* | .411** | .131 | — | .412** |
| 9. FCz CNV CueNoWin | −.103 | −.063 | −.173 | −.135 | −.067 | .206* | .501** | .412** | — |

**Note.** N = 103 for CNV and Age; N = 101 for FRN; N = 96 for RewP.

- p < .05. ** p < .01 (two-tailed).

Table S4 - Regression analysis

To which extent EEG reward-related activity can explain AD symptoms

| Coefficients^a^ | | | | | | | | |
| --- | --- | --- | --- | --- | --- | --- | --- | --- |
| Model | | Unstandardized Coefficients | | Standardized Coefficients | t | Sig. | Collinearity Statistics | |
|  |  | B | Std. Error | Beta |  |  | Tolerance | VIF |
| 1 | (Constant) | .962 | .201 |  | 4.787 | .000 |  |  |
|  | Sex | -.222 | .127 | -.176 | -1.745 | .084 | .941 | 1.063 |
|  | Medication | .513 | .206 | .251 | 2.488 | .015 | .941 | 1.063 |
| 2 | (Constant) | 1.026 | .195 |  | 5.247 | .000 |  |  |
|  | Sex | -.233 | .124 | -.185 | -1.882 | .063 | .928 | 1.077 |
|  | Medication | .554 | .200 | .271 | 2.773 | .007 | .936 | 1.069 |
|  | Cz_CNV_Cue Monetary | -.001 | .014 | -.006 | -.061 | .952 | .827 | 1.209 |
|  | FCZ_CNV_Cue Monetary | .042 | .016 | .283 | 2.728 | .**008** | .830 | 1.205 |
| 3 | (Constant) | 1.008 | .194 |  | 5.184 | .000 |  |  |
|  | Sex | -.227 | .125 | -.180 | -1.822 | .072 | .895 | 1.118 |
|  | Med | .554 | .199 | .271 | 2.785 | .007 | .926 | 1.080 |
|  | Cz_CNV_Cue Monetary | -.007 | .014 | -.050 | -.473 | .637 | .786 | 1.272 |
|  | FCZ_CNV_Cue Monetary | .040 | .016 | .270 | 2.560 | .**012** | .791 | 1.264 |
|  | RewP_Monetary | -.028 | .015 | -.187 | -1.815 | .073 | .830 | 1.205 |
|  | RewP_Verbal | .015 | .014 | .115 | 1.136 | .259 | .857 | 1.167 |
| a. Dependent Variable: DADYS – Parents total scale | | | | | | | | |

Model 1: F(2,94)=6.029, p=.003

Model 2: F(4,94)=5.423, p=.001

Model 3: F(6,94)=4.306, p=.001

Table S5 - Additional regression analysis including ADHD, Aggression and Anxiety/Depressive symptoms

| Model Summary | | | | | | | | | |  |
| --- | --- | --- | --- | --- | --- | --- | --- | --- | --- | --- |
| Model | R | R Square | Adjusted R Square | Std. Error of the Estimate | Change Statistics | | | | |  |
|  |  |  |  |  | R Square Change | F Change | df1 | df2 | Sig. F Change |  |
| 1 | .340^a^ | 0.116 | 0.097 | 0.5986750 | 0.116 | 6.029 | 2 | 92 | 0.003 |  |
| 2 | .890^b^ | 0.793 | 0.781 | 0.2945494 | 0.677 | 97.020 | 3 | 89 | 0.000 |  |
| 3 | .900^c^ | 0.810 | 0.795 | 0.2852885 | 0.017 | 3.936 | 2 | 87 | 0.023 |  |
| 4 | .902^d^ | 0.813 | 0.793 | 0.2864472 | 0.003 | 0.649 | 2 | 85 | 0.525 |  |
| a. Predictors: (Constant), Medication, Sex | | | | | | | | | |  |
| b. Predictors: (Constant), Medication, Sex, CBCL Anxiety/Depressive - T1, SCL-ADHD Total scale ADHS - T1, CBCL Aggressive behavior - T1 | | | | | | | | | |  |
| c. Predictors: (Constant), Medication, Sex, CBCL Anxiety/Depressive - T1, SCL-ADHS Total scale - T1, CBCL Aggressive behavior - T1, Cz_CNV_CueWin, FCZ_CNV_CueWin | | | | | | | | | |  |
| d. Predictors: (Constant), Medication, Sex, CBCL Anxiety/Depressive - T1, SCL-ADHS Total scale - T1, CBCL Aggressive behavior - T1, Cz_CNV_CueWin, FCZ_CNV_CueWin, RewP_Verbal, RewP_Monetary | | | | | | | | | |  |
| e. Dependent Variable: DADYS-Parent Total scale - T1 | | | | | | | | | |  |

| Coefficients ^a^ | | | | | | | | |
| --- | --- | --- | --- | --- | --- | --- | --- | --- |
| Model | | Unstandardized Coefficients | | Standardized Coefficients | t | Sig. | Collinearity Statistics | |
|  |  | B | Std. Error | Beta |  |  | Tolerance | VIF |
| 1 | (Constant) | 0.962 | 0.201 |  | 4.787 | 0.000 |  |  |
|  | Sex | -0.222 | 0.127 | -0.176 | -1.745 | 0.084 | 0.941 | 1.063 |
|  | Medication | 0.513 | 0.206 | 0.251 | 2.488 | 0.015 | 0.941 | 1.063 |
| 2 | (Constant) | 0.136 | 0.113 |  | 1.207 | 0.231 |  |  |
|  | Sex | -0.015 | 0.065 | -0.012 | -0.232 | 0.817 | 0.865 | 1.157 |
|  | Medication | 0.189 | 0.106 | 0.093 | 1.794 | 0.076 | 0.869 | 1.150 |
|  | SCL-ADHS Total scale - T1 | 0.030 | 0.068 | 0.032 | 0.442 | 0.659 | 0.453 | 2.207 |
|  | CBCL Aggressive behavior- T1 | 1.054 | 0.106 | 0.739 | 9.895 | 0.000 | 0.417 | 2.396 |
|  | CBCL Anxiety/Depressive- T1 | 0.315 | 0.118 | 0.160 | 2.676 | 0.009 | 0.650 | 1.539 |
| 3 | (Constant) | 0.186 | 0.111 |  | 1.682 | 0.096 |  |  |
|  | Sex | -0.019 | 0.064 | -0.015 | -0.296 | 0.768 | 0.855 | 1.169 |
|  | Medication | 0.214 | 0.103 | 0.105 | 2.088 | 0.040 | 0.863 | 1.159 |
|  | SCL-ADHS Total scale - T1 | 0.031 | 0.066 | 0.033 | 0.471 | 0.639 | 0.451 | 2.219 |
|  | CBCL Aggressive behavior- T1 | 1.055 | 0.104 | 0.739 | 10.186 | 0.000 | 0.414 | 2.413 |
|  | CBCL Anxiety/Depressive- T1 | 0.240 | 0.118 | 0.122 | 2.039 | 0.044 | 0.612 | 1.634 |
|  | Cz_CNV_Cue Monetary | 0.000 | 0.007 | 0.001 | 0.023 | 0.982 | 0.817 | 1.225 |
|  | FCZ_CNV_Cue Monetary | 0.020 | 0.008 | 0.136 | 2.550 | **0.013** | 0.764 | 1.309 |
| 4 | (Constant) | 0.196 | 0.112 |  | 1.755 | 0.083 |  |  |
|  | Sex | -0.026 | 0.065 | -0.021 | -0.398 | 0.692 | 0.821 | 1.219 |
|  | Medication | 0.225 | 0.104 | 0.110 | 2.168 | 0.033 | 0.848 | 1.179 |
|  | SCL-ADHS Total scale - T1 | 0.023 | 0.068 | 0.024 | 0.337 | 0.737 | 0.438 | 2.285 |
|  | CBCL Aggressive behavior- T1 | 1.060 | 0.104 | 0.743 | 10.145 | 0.000 | 0.410 | 2.436 |
|  | CBCL Anxiety/Depressive- T1 | 0.226 | 0.120 | 0.114 | 1.877 | 0.064 | 0.591 | 1.691 |
|  | Cz_CNV_Cue Monetary | -0.002 | 0.007 | -0.013 | -0.247 | 0.806 | 0.768 | 1.302 |
|  | FCZ_CNV_Cue Monetary | 0.021 | 0.008 | 0.142 | 2.605 | **0.011** | 0.736 | 1.358 |
|  | RewP_Monetary | -0.006 | 0.008 | -0.038 | -0.707 | 0.481 | 0.783 | 1.278 |
|  | RewP_Verbal | 0.007 | 0.007 | 0.054 | 1.056 | 0.294 | 0.830 | 1.204 |
| a. Dependent Variable: DADYS-Parent Total scale - T1 | | | | | | | | |

Table S6 - Predicting CNV activity

| Predicting CNV activity – Regression analyses | | | | |
| --- | --- | --- | --- | --- |
|  | Reward anticipation CNV at FCz | | |  |
| Predictors | beta | t | p | Model p |
| DADYS Irritability/Impulsivity | .249 | 2.360 | .020 | .096 |
| DADYS Promis (Anger/Irritability) | .299 | 2.947 | **.004** | **.026** |
| DADYS Exhuberance | .238 | 2.317 | .023 | .095 |
| DADYS Function | .246 | 2.254 | .027 | .114 |
| DADYS Positive emotionality | -102 | -.925 | .357 | .624 |
| CBCL Aggression | .194 | 1.799 | .075 | .250 |
| SCL-ADHD | .241 | 2.267 | .026 | .115 |
| CBCL Anxiety/Depression | .301 | 3.064 | **.003** | **.019** |
|  | Reward delivery RewP | |  |  |
| DADYS Irritability/Impulsivity | -.136 | -1.242 | .218 | .298 |
| DADYS Promis (Anger/Irritability) | -.147 | -1.379 | .171 | .258 |
| DADYS Exhuberance | -.204 | -1.950 | .054 | .153 |
| DADYS Function | -.231 | -2.062 | .042 | 0.96 |
| DADYS Positive emotionality | .120 | 1.077 | .284 | .322 |
| DADYS Total score | -.134 | -1.234 | .221 | .301 |
| CBCL Aggression | -.182 | -1.688 | .095 | .175 |
| SCL-ADHD | -.086 | -.775 | .440 | .433 |
| CBCL Anxiety/Depression | -.259 | -2.581 | .011 | .035 |
|  | Reward delivery FRN | |  |  |
| DADYS Irritability/Impulsivity | .001 | .012 | .990 | .571 |
| DADYS Promis (Anger/Irritability) | -.045 | -.419 | .676 | .535 |
| DADYS Exhuberance | -.079 | -.750 | .455 | .440 |
| DADYS Function | .002 | .016 | .988 | .495 |
| DADYS Positive emotionality | -.015 | -.134 | .893 | .510 |
| DADYS Total score | -.003 | -.026 | .979 | .571 |
| CBCL Aggression | -.001 | -.010 | .992 | .571 |
| SCL ADHD | -002 | -019 | -985 | -571 |
| CBCL Anxiety/Depression | .027 | .260 | .796 | .557 |
|  |  |  |  |  |

| In Bold: CBCL Anxiety/Depression scale and the DADYS Promis (Anger/Irritability) |
| --- |

Significantly predicted (after correction for multiple testing) reward anticipation deficits measured at FCz.

Figure S1 – Reward anticipation task


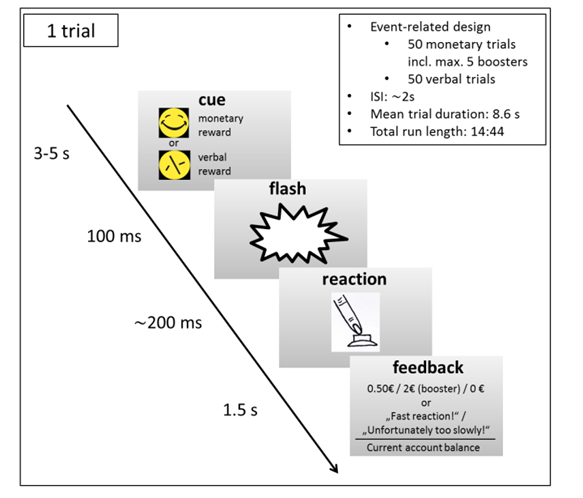


The reward anticipation task requires a fast button press to a flash cued by a smiley or a scrambled smiley indicating monetary or verbal feedback.

Figure S2 - Reaction time


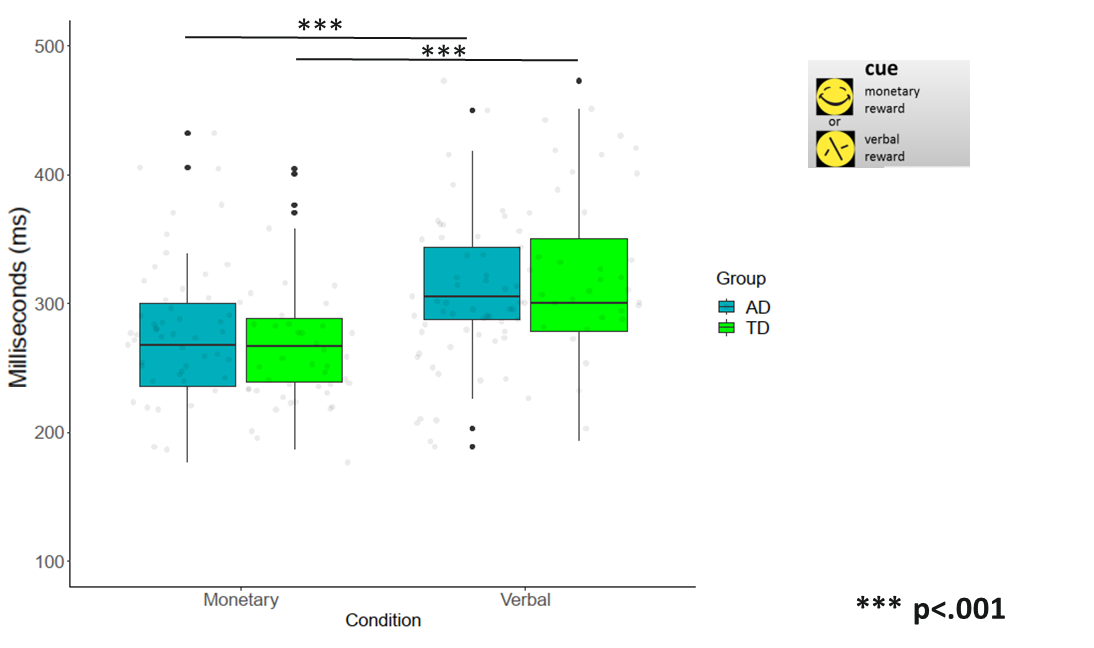


Appendix S1 – Sensitivity analysis

Controlling for site and medication

Anticipation phase: After including those additional covariates the significant group x condition interaction remained [F(1,96)=7.470, p=0.007, part. η2=0.072] and revealed also that the AD group had reduced anticipatory CNV amplitude. Post-hoc test showed that this between-group difference was stronger for the cue monetary condition (monetary cue: p<.001, verbal cue: p=.655), and that only the No-AD group showed a significant difference between conditions (p<0.001). The group x channel interaction remained significant [F(1,96)=5.985, p=0.016, part. η2=0.059]. Post-hoc tests revealed that the group differences were also more pronounced at the fronto-central electrode (FCz, p=.004).

Delivery phase: No significant main effect or interaction emerged neither for RewP or FRN (all p>.121), but the AD group tended to show a less pronounced RewP at the selected fronto-central electrodes for the monetary condition (p=.079). Medication showed to have a significant impact on FRN (p=0.011).
